# Evaluation of Alpha-Amylase Inhibitory, Antioxidant, and Antimicrobial Potential and Phytochemical Contents of *Polygonum hydropiper* L.

**DOI:** 10.3390/plants9070852

**Published:** 2020-07-06

**Authors:** Abdul Nasir, Mushtaq Khan, Zainab Rehman, Atif Ali Khan Khalil, Saira Farman, Naeema Begum, Muhammad Irfan, Wasim Sajjad, Zahida Parveen

**Affiliations:** 1 Department of Biochemistry, Abdul Wali Khan University Mardan, Mardan 23200, Pakistan; anasirqau@gmail.com (A.N.); mushtaqkhanbiochemist@gmail.com (M.K.); sairafarman@awkum.edu.pk (S.F.); yzai_taurus@yahoo.com (N.B.); 2 Department of Molecular Science and Technology, Ajou University, Suwan 16499, Korea; 3 Laboratory of Animal and Human Physiology, Department of Animal Sciences, Quaid-i-Azam University, Islamabad 45320, Pakistan; abbassids@outlook.com; 4 Department of Biological Sciences, National University of Medical Sciences, Rawalpindi 46000, Pakistan; atif.ali@numspak.edu.pk; 5 College of Dentistry, Department of Oral Biology, University of Florida, Gainesville, FL 32610, USA; irfanmuhammad@ufl.edu

**Keywords:** water-pepper, FRAP, DPPH, polarity-based solvent extraction, α-amylase inhibition

## Abstract

*Polygonum hydropiper* L. is a traditionally used medicinal plant. The present study was designed to explore the α-amylase inhibitory, antioxidant, and antimicrobial activities of *Polygonum hydropiper* L. Polarity-based solvent extracts (*n*-hexane, acetone, chloroform, methanol, ethanol, and water) of *Polygonum hydropiper* leaves and stem were used. Antioxidant activity was assessed by free radical scavenging assay (FRAP) and 2,2-diphenylpicrylhydrazyl (DPPH) free radical scavenging activity methods. Quantitative phytochemical analyses suggested that the stem of *Polygonum hydropiper* L. contains higher levels of bioactive compounds than its leaves (*p* < 0.05). The results suggested that stem-derived extracts of *Polygonum hydropiper* L. are more active against bacterial species, including two Gram-positive and three Gram-negative strains. Moreover, our results showed that the bioactive compounds of *Polygonum hydropiper* L. significantly inhibit α-amylase activity. Finally, we reported the polarity-based solvent extracts of *Polygonum hydropiper* L. and revealed that the stem, rather than leaves, has a high antioxidant potential as measured by FRAP and DPPH assay with IC_50_ values of 1.38 and 1.59 mg/mL, respectively. It may also be deducted from the data that the *Polygonum hydropiper* L. could be a significant candidate, which should be subjected to further isolation and characterization, to be used as an antidiabetic, antimicrobial and antioxidant resource in many industries, like food, pharmaceuticals and cosmetics.

## 1. Introduction

The Polygonaceae family is comprised of 48 genera and 1200 species [1,2]. Among the 60 species of the *Polygonum* distributed throughout the world, approximately 20 species are found in Pakistan [3]. They grow in moist places and shallow water. *Polygonum hydropiper* L. is an important medicinal plant of Polygonaceae family. It is commonly grown annually in the wet areas and is recognized as a common weed which is native to Southeast Asia [4]. Morphologically, the stem is round; its length usually varies from 40 to 70 cm [5].

This species has anti-inflammatory potential [6] and insecticidal properties [7], along with anticholinesterase, phytotoxic, anthelmintic, antiangiogenic, anticancer, antimicrobial, and antioxidant potential [8,9,10,11]. This plant is used in treating broad range of disorders, including gastrointestinal disturbances, neurological disorders, inflammation, and diarrhea [12]. Moreover, it is used to treat other diseases, like dyspepsia, itching skin, excessive menstrual bleeding, hemorrhoids, and cancer (particularly colon, breast, and prostate cancer) [13]. Furthermore, sap of the leaves is used to treat headache, pain, toothache, liver enlargement, gastric ulcer, dysentery, and loss of appetite. The juice of this plant is considered useful for treating wounds, skin diseases, and painful carbuncles [14]; it can also be used as an anthelminthic herb, in the treatment of snake-bites, and as a diuretic [15].

The phytochemicals of *P. hydropiper* L. include catechins, procyanidins, condensed tannins, and antitumor agents that are flavanols in nature [16]. Flavonoids with antioxidant activity and aldose reductase and tyrosinase inhibitory activities have also been found in *P*. *hydropiper* L. [4,17]. Due to the presence of drimane-type sesquiterpenes, *P*. *hydropiper* L. has some insecticidal and antifungal activities [18,19]. The antifungal activities of *P*. *hydropiper* L. are due to the presence of confertifolin, a natural antimicrobial compound [20]. Other bioactive compounds of *P*. *hydropiper* L. include gallic acid; ellagic acid; 3,3′-di-O-methyl ether; anthraquinone; aromatic 6-lactone; and flavonoids such as viscosumic acid, oxymethyl-anthraquinones, rutin, hyperin, isoquercitrin, epicatechin, quercetin, kaempferol, and isorhamnetin [21]. It has been reported that this species contains pyrocatechol, 4-methyloxazole, caryophyllene, succinimide, vanillic acid, myristic acid, farnesol, arachidic acid, methyl ester, and capsaicin [10].

The excessive and long-term use of synthetic drugs causes several side effects [22]. In the human body, numerous cellular processes produce free radicals (FR) and reactive oxygen species (ROS). Such toxics may also be produced exogenously by pollutants, radiation, smoke, drugs, and xenobiotics [23]. Overproduction of different chemically reactive oxygen plagiaristic molecules such as hydrogen peroxide (H_2_O_2_), superoxide (O_2_^−^), and hydroxyl radicals (OH^−^) is highly toxic and leads to biomolecular damage, which in turn causes diabetes mellitus, cancer, atherosclerosis, and heart and neurodegenerative diseases [24]. In contrast, the overproduction of FR and ROS species can be averted by antioxidant substances. It is reported that secondary metabolites of plants like phenols, flavonoids, and alkaloids have antioxidant, antidiabetic, anthelminthic, lipid-lowering, anticoagulant, and antimicrobial properties [25].

Diabetes mellitus is characterized by hyperglycemia, lipedema, and oxidative stress and predisposes affected individuals to long-term complications afflicting the eyes, skin, kidneys, nerves, and blood vessels. Recently, it has been estimated that the prevalence of diabetes by 2025 will increase from 143 million to 300 million patients [26]. Various studies have indicated that dietary supplementation with combined antiglycation and antioxidant nutrients might be a safe and simple complement to traditional therapies targeting diabetic complications [27]. Hyperglycemia, through both enzymatic and non-enzymatic mechanisms, produces oxidative stress by producing free radicals. α-Amylase hydrolyzes (1,4)-α-D-glucosidic linkages in polysaccharides containing three or more (1,4)-α-linked D-glucose units, which ultimately increases the blood sugar level; α-amylase-inhibiting drugs such as acarbose, miglitol, and voglibose are frequently used in diabetes management [28,29]. However, α-amylase-inhibiting drugs cause severe side effects like bloating and abdominal uneasiness [30]. Therefore, the administration of natural resources to treat diabetes seems to be a promising strategy. To this end, targeting α-amylase inhibition by natural remedies may be an ideal platform in diabetes prevention [31,32]. In this context, the current study was designed to determine the bioactive compounds of *P. hydropiper*. Furthermore, the extracts of *P. hydropiper* were evaluated for their potential to inhibit α-amylase activity and reduce oxidative stress. Finally, the antimicrobial potential of *P*. *hydropiper* was demonstrated.

## 2. Results

### 2.1. Determination of Bioactive Compounds

In first series of experiments, class of secondary metabolites including alkaloids, tannins, flavonoids, β-carotene, and lycopene from the leaves (PHL) and stem (PHS) of *P. hydropiper* were extracted with their respective solvents and quantified. The comparative analyses found that concentrations of alkaloids and tannins were significantly higher in *P*. *hydropiper* stem than in leaves (t = −3.22, *p* = 0.032; t = −3.61, *p* = 0.023). However, flavonoids were found in comparable amounts in both stem and leaves (t = −0.427, *p* = 0.691) (Table 1). We also noticed that the leaves of *P. hydropiper* contained significantly higher concentrations of β-carotene and lycopene than those found in the stem of *P. hydropiper* (t = 2.90, *p* = 0.044; t = 4.31, *p* = 0.013).

### 2.2. In Vitro Evaluation of α-Amylase Inhibition

**Figure 1:**
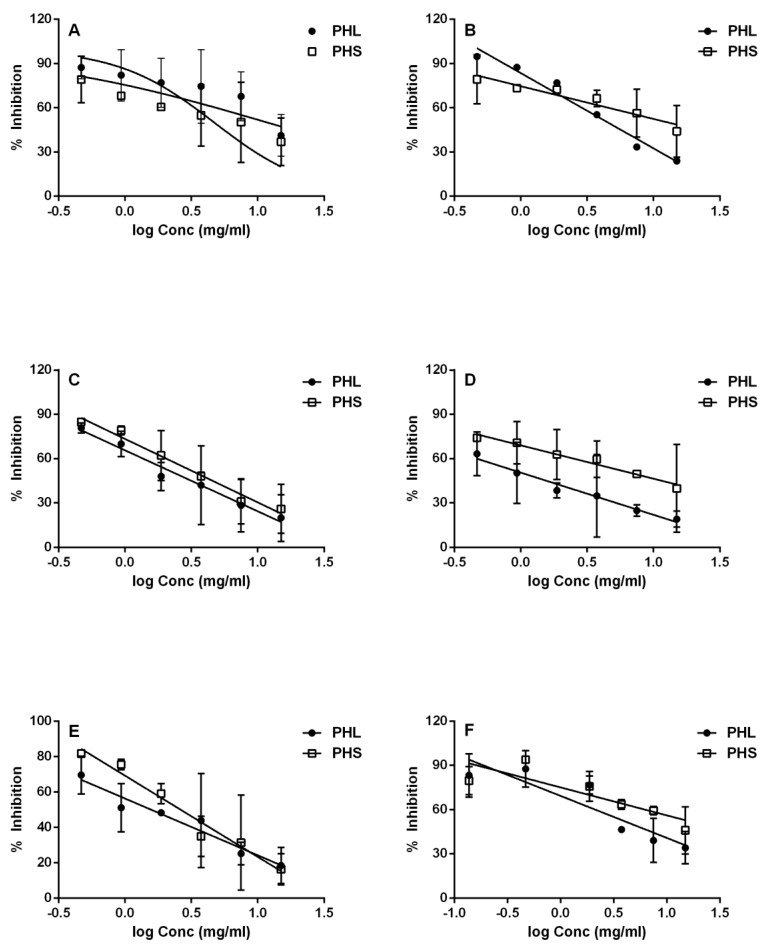
Inhibition of α-amylase activity by different extracts (ethanol, (**A**); acetone, (**B**); methanol, (**C**); *n*-hexane, (**D**); chloroform, (**E**) and water, (**F**)) of *P. hydropiper* leaves (PHL) and stem (PHS). Solid lines represent hyperbolic dose–response curves, which were generated in GraphPad Prism. Values represent the means of triplicate measurements (n = 3).

**Figure 2:**
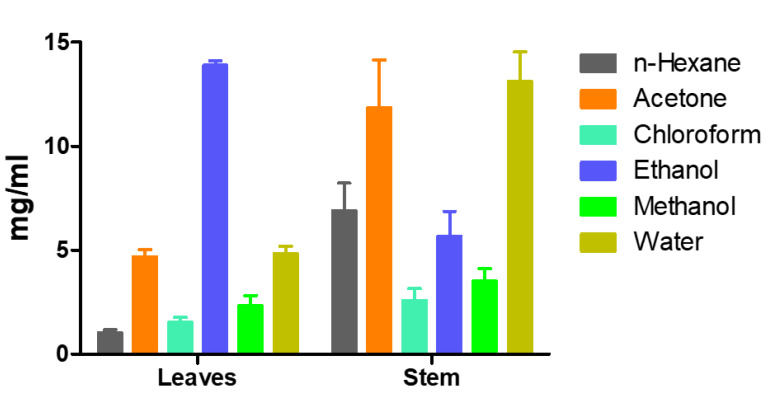
α-Amylase inhibitory activity of the different extracts tested: comparison of IC_50_ values. The IC_50_ values were calculated from dose-dependent percent inhibition. Values represent the means of triplicate measurements (n = 3). Bars represent the standard deviation.

The different solvent extracts of *P. hydropiper* stem and leaves were investigated for their potential to inhibit α-amylase activity at six different concentrations (0.46, 0.94, 1.88, 3.75, 7.50, and 15 mg/mL). The dose–response calibration curves for *n*-hexane, acetone, chloroform, ethanol, methanol, and water extracts of *P. hydropiper* leaves and stem were constructed individually. Percent α-amylase inhibition and IC_50_ values were determined from the dose–response calibration curves for each type of extract (Figure 1 and Figure 2).

The α-amylase inhibitory activities of the leaf extracts were ranked in the following order: *n*-hexane (IC_50_ 1.03 mg/mL; R^2^ 0.566) ˃ chloroform (IC_50_ 1.53 mg/mL; R^2^ 0.6492) ˃ methanol (IC_50_ 2.32 mg/mL; R^2^ 0.7255) ˃ acetone (IC_50_ 4.70 mg/mL; R^2^ 0.9919) ˃ water (IC_50_ 4.85 mg/mL; R^2^ 0.7629) ˃ ethanol (IC_50_ 13.89 mg/mL; R^2^ 0.3249). Differently from those of the leaf extracts, the α-amylase inhibitory activities of the stem extracts were ranked in the following order: chloroform (IC_50_ 2.599 mg/mL; R^2^ 0.8232) ˃ methanol (IC_50_ 3.517 mg/mL; R^2^ 0.8375) ˃ ethanol (IC_50_ 5.672 mg/mL; R^2^ 0.4736) ˃ *n*-hexane (IC_50_ 6.910 mg/mL; R^2^ 0.4399) ˃ acetone (IC_50_ 11.86 mg/mL; R^2^ 0.5608) ˃ water (IC_50_ 13.12 mg/mL; R^2^ 0.6824).

### 2.3. Antioxidant Capacity

The antioxidant potential of the different extract types was analyzed at six concentrations (0.46, 0.94, 1.88, 3.75, 7.50, and 15 mg/mL) by using the free radical scavenging assay (FRAP) and the 2,2-diphenylpicrylhydrazyl (DPPH) assay. Table 2 shows the antioxidant potential measured by two assays.

The FRAP activities for different extract types of both stem (PHS) and leaves (PHL) were observed to be ranked in the following order: PHS ethanol (IC_50_ 1.38 mg/mL; R^2^ 0.7847) > PHS *n*-hexane (IC_50_ 1.50 mg/mL; R^2^ 0.8407) > PHS methanol (IC_50_ 1.73 mg/mL; R^2^ 0.8483) > PHS acetone (IC_50_ 1. 81 mg/mL; R^2^ 0.8814) > PHL acetone (IC_50_ 2.29 mg/mL; R^2^ 0.977) > PHL methanol (IC_50_ 2.30 mg/mL; R^2^ 0.9121) > PHL ethanol (IC_50_ 2.99 mg/mL; R^2^ 0.8090) > PHL n-hexane (IC_50_ 5.52 mg/mL; R^2^ 0.7588). All types of extracts of the stem showed higher FRAP activity than those of the leaves of *P*. *hydropiper*.

The DPPH activities of both stem and leaves were also analyzed for different extract types at different concentrations. The acetonic extracts of both stem and leaves were more effective than ethanolic extracts. The activities was observed to be ranked in the following order: PHS acetone (IC_50_ 1.59 mg/mL; R^2^ 0.8330) > PHL acetone (IC_50_ 2.94 mg/mL; R^2^ 0.9244) > PHL ethanol (IC_50_ 5.14 mg/mL; R^2^ 0.9102) > PHS ethanol (IC_50_ 6.88 mg/mL; R^2^ 0.8126).

### 2.4. Antimicrobial Activity

Our results showed strong antibacterial activities for both acetonic and ethanolic extracts at all six concentrations (0.46, 0.94, 1.88, 3.75, 7.50, and 15 mg/mL) of *P. hydropiper* stem and leaves. Zones of inhibition of the tested extracts are depicted in Table 3. The acetonic stem extract showed antibacterial activity against five different microbial species (*Escherichia coli*, *Staphylococcus aurous*, *Klebsiella pneumoniae*, *Morganella morganii*, and *Haemophilus influenzae*); however, the leaves did not show antibacterial activity against *E. coli and S. aureus*. The ethanolic extracts of stem and leaves showed strong antiproliferative activities against all the microbial species tested (Table 3).

## 3. Discussion

The excessive use of synthetic drugs causes several side effects and may lead to conflict [22]. In contrast, traditional medicines are harmless, effective, and inexpensive drug candidates. In order to evaluate the biological activities of plant extracts with respect to traditional uses, *P. hydropiper* was previously screened for its antioxidant potency and antimicrobial and antipathogenic activities at low doses and with few solvent extractions [8,10]. We evaluated the antioxidant and antibacterial potential at high doses and with several different solvent extractions; additionally, we screened the α–amylase inhibitory potential of *P. hydropiper*.

Our results show that *P. hydropiper* leaves are rich in tannins and flavonoids, which is in strong agreement with the results of Nakao et al. (1999), who they reported condensed tannins, procyanidins, and catechins which are flavones in nature [16]. Yang et al. (2011) also reported the presence of flavonoid in *P. hydropiper* leaves [21]. Previously, it was reported that *P. hydropiper* species contained 4-methyloxazole (flavonoid in nature) and succinimide [8]. The therapeutic application of β-carotene showed a strong association with a lower risk of lung cancer [33]. β-Carotene along with phenytoin has great antiepileptic activity and can be used as a therapeutic agent in epilepsy management [34]. Tannins have been reported as anticancer and anti-inflammatory agents and can be used for ulcerated tissues [35,36,37]. Polyphenols are known not only to ease the oxidative stress status, but also to act on cellular signaling pathways, including vascular endothelial growth factor (VEGF)-mediated angiogenesis, endoplasmic reticulum (ER) stress, nitric oxide (NO^∙^) signaling, and nuclear factor E2-related factor 2 antioxidant pathways, thus preventing vascular complications in diabetes. Resveratrol, one of the most studied polyphenols, has been reported to restore the insulin receptor substrate 1 (IRS-1) and endothelial nitric oxide synthase (eNOSx) signaling pathway in endothelial cells under palmitate-induced insulin resistance [38,39]. As a source of tannins, *P. hydropiper* may be used to treat cancer, inflammation, and ulcerated tissues. Furthermore, our findings revealed that the leaves of *P. hydropiper* contain a significantly high level of lycopene. The intake of lycopene reduces the risk of prostate cancer [40] and plays a protective role against nephrotoxicity [41]. Lycopene consumption can also regulate endothelial function, thereby reducing oxidative stress in healthy humans [42].

Enzymes that are primarily involved in increasing blood glucose, such as α-amylase and α-glucosidase, have been targeted as a therapeutic approach in postprandial hyperglycemia [43] because their inhibition can cause reduction in postprandial hyperglycemia [44]. Our results indicate that α-amylase activity was inhibited by the extracts of *P. hydropiper* leaves only. Specifically, the *n*-hexane extract of the leaves showed the most potent antiamylase activity. Therefore, we suggest that leaf extracts of *P. hydropiper* could be used as a future therapeutic candidate in treating hyperglycemia.

The antioxidant activity of leaf and stem extracts was determined by two methods: the free radical scavenging assay (using ferric ion reducing agent) using and the DPPH scavenging assay. The results in our study indicate that this plant species was potently active, which suggests that all different extracts of *P. hydropiper* leaves and stem contained compounds that are capable of hydrogen donation to the free radical for the purpose of eliminating the odd electron, thus reducing the radicals’ activity. This also implies that this plant species may be beneficial, especially at high concentrations, for treating the pathological damage caused by radicals’ activities. All of the extracts of both stem and leaves at different concentrations were also active against the Gram-negative and Gram-positive bacteria. However, such activity was not shown by the acetonic extract of leaves against *E. coli* (which might be due to outer membrane in Gram-negative bacteria acting as a permeability barrier) and *S. aureus* [45]. Hence, we suggest that ethanolic extracts of *P. hydropiper* stem and leaves could be used as antioxidant and antibacterial agents and should be studied further.

## 4. Materials and Methods

### 4.1. Chemical and Reagents

Soluble starch, potassium ferrocyanide, trichloroacetic acid, ferric chloride, and solvents used for polarity-based extraction (n-hexane, acetone, chloroform, ethanol, and methanol) were purchased from Sigma-Aldrich (Lahore, Pakistan). Dimethylsulfoxide, quercetin, Folin’s phenol, and tannic acid were obtained from Merck (Karachi, Pakistan). Porcine pancreatic α-amylase, ascorbic acid, and DPPH solution were purchased from Sigma-Aldrich (Lahore, Pakistan) for measurement of α-amylase, FRAP, and DPPH assay, respectively. All reagents were biochemical reagent grade.

### 4.2. Sample Collection

*Polygonum hydropiper* was collected from Mardan District, Khyber Pakhtunkhwa, Pakistan, in April 2016. The plant was identified as *P. hydropiper* and deposited in the Department of Botany, Abdul Wali Khan University, Mardan, Pakistan.

### 4.3. Sample Preparation

Fresh leaves and stem of *P. hydropiper* were separated, washed with tap water to remove dust, and then air-dried at 25 °C for 30 days in an air flux drying oven. The plant parts were crushed to fine powder (80 mashes) with the help of an electric blender. Powdered samples were placed in sterile sealed bags each containing a damp paper towel and kept at 4 °C for further analyses.

### 4.4. Solvent–Solvent Extraction

The extracts of powdered samples were isolated using the solvent–solvent extraction method. Several solvents (n-hexane, acetone, chloroform, ethanol, methanol, and water) were used to ensure the polarity-based extraction. Initially, each sample was extracted by shaking with n-hexane at a ratio of 1:10 (w/v) for 24 h at room temperature. The corresponding sample was then filtered by Whatman filter paper. The pellet was separated, and the supernatant was transferred to a preweighed Falcon tube. The residual pellet was re-extracted with next solvent, which was slightly polar than n-hexane, using the same ratio, temperature, and time. The same procedure was repeated with all solvents, and all extracts were allowed to dry in oven at 37 °C. The dried sample was weighed and redissolved in dimethylsulfoxide (DMSO) at a final concentration of 15 mg/mL.

### 4.5. Phytochemical Determination

#### 4.5.1. Determination of Flavonoids

Flavonoid estimation was carried out by spectrophotometric assay as previously described [46]. Five grams of air-dried powdered sample was dissolved in 50 mL of 80% aqueous ethanol and incubated for 24 h in a shaker incubator. The extract was centrifuged at 10,000 rpm and 25 °C for 15 min. The pellet was discarded and the supernatant containing flavonoids was stored in a 50 mL Falcon tube at 4 °C. The flavonoid extract (250 μL) was mixed with 1.25 mL of sterile distilled water and 75 μL of 5% NaNO_2_ solution. After 5 min, 150 μL of 10% AlCl_3_.H_2_O was added, and the mixture was incubated for 6 min. Thereafter, 500 μL of 1 M NaOH and 275 μL of sterile distilled water were added to the mixture. The solution was mixed, and absorbance was measured at λ of 415 nm. Different concentrations of quercetin (15–500 μg) were used as standards to calculate the standard curve, while 80% aqueous ethanol was used as blank.

#### 4.5.2. Determination of β-Carotene and Lycopene

β-Carotene and lycopene were extracted and quantified according to the method described by Lillian et al. [47]. Briefly, methanol extract was prepared by dissolving 10 g of air-dried powdered sample in 100 mL of methanol and incubating in a shaker incubator for 24 h. The extract was filtered by Whatman filter paper, the pellet was discarded, and supernatant was isolated. Thereafter, the methanol contents were evaporated by heating in a water bath. The dried sample was dissolved in acetone and n-hexane mixture (4:6). Finally, the reaction mixture containing β-carotene and lycopene was stored at 4 °C. The spectrophotometric analysis was carried out by measuring absorbance at λ’s of 453, 505, 645, and 663 nm. β-Carotene and lycopene contents were calculated [48] by using the following equations:

(1) $$\mathrm{Lycopene}\;\left(\mathrm{mg}/50\;\mathrm{mL}\right)=0.0458A_{663}+0.372A_{505}-0.0806A_{453}$$

(2) $$\beta -\mathrm{carotene}\;(\mathrm{mg}/50\;\mathrm{mL})=0.216A_{663}+0.304A_{505}-0.452A_{453}$$

#### 4.5.3. Determination of Tannins

Tannin contents of *P. hydropiper* were extracted using the method of Makkar et al. [49]. Briefly, 0.5 g of each air-dried powdered sample was dissolved in 100 mL of 70% acetone and incubated while shaking for 6 h. The sample was filtered by Whatman filter paper, the pellet was discarded, and the supernatant was stored in a 50 mL Falcon tube at 4 °C. Different concentrations of tannic acid (3–50 mg) were prepared by serial dilution from stock solution (50 mg/100 mL of 70% acetone). The tannin extracts (50 μL) was mixed with 950 μL of sterile distilled water. Thereafter, 0.5 mL of Folin’s phenol reagent (mixture of phosphomolybdate and phosphotungstate), used for phenolic and polyphenolic antioxidants detection, and 2.5 mL of 20% NaCO_3_ solution were added and vortexed. The solution was incubated at room temperature for 40 min. Finally, the absorbance was measured at λ of 725 nm. During the experiment, 70% acetone was used as blank and treated as positive control.

#### 4.5.4. Determination of Alkaloids

Alkaloids were extracted by using the acid–base shifting method [46]. Briefly, dried powdered sample was dissolved in ethanol at a ratio of 1:10 (w/v) and left to shake for 24 h at room temperature. The extract was concentrated while drying in an oven. The dried sample was redissolved in ethanol with 1% HCl. The mixture was then precipitated in a refrigerator for 3 days. The solution was filtered, and pH was maintained at 8–10 by the addition of ammonium hydroxide. This basic solution was extracted with chloroform by using a separating funnel. The chloroform layer containing alkaloids was recovered, and ethanol layer was discarded. The chloroform was evaporated by heating in a water bath. Finally, the sample was dried in an oven and alkaloid contents were measured.

### 4.6. α-Amylase Inhibition Assay

Screening of extracts for α-amylase inhibition was carried as previously described for the starch iodine assay [5]. In brief, the assay mixtures composed of 120 μL of 0.02 M sodium phosphate buffer (pH 6.9 containing 6 mM sodium chloride), 1.5 mL of porcine pancreatic α-amylase (PPA) solution (0.05 mg/2 mL H_2_O) and *P. hydropiper* extracts (sample from solvent–solvent extraction) at concentrations ranging from 0.46 to 15 mg/mL (w/v) were incubated at 37 °C for 10 min. Afterward, soluble starch (1%, 0.1 g/10 mL (w/v)) was added to each reaction test tube and incubated at 37 °C for 15 min. Then, 1 M HCl (60 μL) was added to stop the enzymatic reaction, followed by the addition of 300 μL of iodine reagent (5 mM I_2_ and 5 mM KI). The color change was noted, and the absorbance was recorded at λ of 620 nm on a spectrophotometer (721 2C50811136 Shimadzu, Japan). The control reaction representing 100% enzyme activity did not contain any *P. hydropiper* extract. To eliminate the absorbance produced by extracts, appropriate extract controls without α-amylase were also included. A dark blue color indicates the presence of starch, a yellow color indicates the absence of starch, and a brownish color indicates partially degraded starch in the reaction mixture. In the presence of extracts, the starch will not degrade upon its addition to the enzyme assay mixture, hence giving a dark blue color complex. In contrast, the absence of color complex indicates the lack of inhibitor and means that starch is completely hydrolyzed by α-amylase. The percentage inhibition of α-amylase was calculated by the following formulas:

(3) $$I_{\alpha -\mathrm{amylase}}\%=\frac{∆A_{\mathrm{control}}-∆A_{\mathrm{sample}}}{∆A_{\mathrm{control}}}\times 100$$

(4) $$∆A_{\mathrm{control}}=A_{\mathrm{test}}-A_{\mathrm{blank}}$$

(5) $$∆A_{\mathrm{sample}}=A_{\mathrm{test}}-A_{\mathrm{blank}}$$

### 4.7. Antioxidant Activity Determination

#### 4.7.1. Free Radical Scavenging Assay (FRAP)

Herein, the ability of extracts to reduce ferric ions was determined by using a previously described method [50], with modifications. The extract (750 μL) of each sample was mixed with an equal amount of phosphate buffer (0.2M, pH 6.6) and 1% potassium ferricyanide (a source of ferric ions). The mixture was incubated at 50 °C for 20 min. After incubation, an equal amount of trichloroacetic acid (10%) was added to stop the reaction. The sample was then centrifuged at 3000 rpm for 10 min. The upper layer (1.5 mL) was separated and mixed with an equal amount of distilled water and 0.1 mL of FeCl_3_ solution (0.1%). A blank was also prepared by using same procedure, and the absorbance was measured at λ of 700 nm as the reducing power. In parallel, ascorbic acid (Vitamin C) was used as standard positive control. The assay was repeated in triplicate, and percentage inhibition was calculated using the following formula:

(6) $$\%\mathrm{Scavenging}\;\mathrm{effect}=\frac{\mathrm{Control}\;\mathrm{absorbance}-\mathrm{Sample}\;\mathrm{absorbance}}{\mathrm{Control}\;\mathrm{absorbance}}\times 100$$

#### 4.7.2. 2,2-Dipheny-1-picrylhydrazyl (DPPH) Scavenging Assay

The DPPH (2,2-dipheny-1-picrylhydrazyl) free radical scavenging capacity of the extract was determined by using a previously described method [51], with modifications. The solution was prepared by dissolving the 0.006 g of DPPH in 100 mL dimethylsulfoxide (DMSO). The extract (1 mL) of each sample was mixed with an equal amount of DMSO and used to prepare desired concentrations of sample (i.e., six concentrations from 0.468 to 15 mg/mL). The solutions of the required concentrations were then transferred into a test tube by taking 1 mL of sample and 2 mL of DPPH solution. The blank was prepared by mixing 1 mL of DMSO with 2 mL of DPPH. All solutions were then incubated for 30 min at 37 °C. The absorbance of each concentration was taken at λ of 517 nm. The percent scavenging activity was calculated as:

(7) $$\%\mathrm{Scavenging}\;\mathrm{activity}=\frac{A_{0}-A_{1}}{A_{0}}\times 100$$

where A_0_ represents absorbance of the control and A_1_ is the absorbance of the sample. Each experiment was performed in triplicate.

### 4.8. Antimicrobial Activity Determination

#### 4.8.1. Selection of Microorganisms

Antimicrobial activity was evaluated against *Escherichia coli*, *Staphylococcus aurous*, *Klebsiella pneumoniae*, *Morganella morganii*, and *Haemophilus influenzae*. Microorganisms were obtained from the National Institute of Health (NIH) in Islamabad, Pakistan. The stock inoculums were subcultured using the streaking method. Inoculums of all microbes were prepared in sterilized LB-broth medium (Miller’s LB Broth, Sigma-Aldrich, St. Louis, MO, USA). Twenty grams of LB-broth powder was added to 1 L of distilled water and autoclaved for 15 min at 121 °C. The autoclaved liquid medium (5 mL) was then poured into separate test tubes and settled to cool at 50 °C. Bacterial inoculums were transferred to the medium-filled test tube and incubated while shaking at 37 °C for 24 h. Later, optical density of each culture was taken at λ of 660 nm and an absorbance level of 0.5–1.0 was considered for the optimal determination of antimicrobial activity.

#### 4.8.2. Preparation of the Culture Medium

The culture medium was prepared by dissolving 20 g of LB-broth medium in 1000 mL of distilled water. A turbid solution was obtained, which was heated until it became a clear transparent solution, using continuous shaking to dissolve the agar completely. The medium was sterilized at 121 °C for 15 min at 15 pounds of pressure. Sterilized medium (35 mL) was poured into petri dishes under the laminar flow hood and left to solidify at room temperature.

#### 4.8.3. Antimicrobial assay

Antimicrobial activity was evaluated by agar well diffusion method. Seventy-five microliters of each microbial culture was spread individually on separate plates. A sterile cork-borer was used to bore wells (9 mm) in each inoculum-spread plate. Acetone and ethanol extracts (100 μL of each) were pipetted into individual wells. In each plate, a negative control (DMSO 100%) and a positive control (ampicillin 10 µg) were treated as standard. The plates were incubated at 37 °C for 24 h. For each plate, zones of inhibition were measured in millimeters.

### 4.9. Statistics

All the statistical analyses were performed using IBM SPSS Statistics 20.0 software (Armonk, NY, USA). The graphs were created in GraphPad Prism 5.0 software (La Jolla, California, USA). One-way ANOVA test was performed for the comparison of groups, and an independent samples t-test was performed for differences between the two groups. All the data are presented as mean and standard error of triplicate measurements. The IC_50_ values were calculated from dose-dependent percent inhibition using GraphPad Prism. Statistically significant difference was considered for *p* < 0.05.

## 5. Conclusions

The present study highlights that *P. hydropiper* possesses a strong α-amylase inhibitory potential and reveals its potency to be used as a strong source of future therapeutic agents in diabetes. Our study also indicates that this plant species may be beneficial for treating the pathological damage caused by radicals’ activities and bacterial infections. Future studies are required to unveil the novel bioactive compounds of *P. hydropiper*, which might be helpful in studying the precise mechanisms of α-amylase inhibition, antioxidant potential, and antimicrobial activity.

## Figures and Tables

**Table 1:** Phytochemical constituents of *P. hydropiper* leaves and stem.

Phytochemical	PHL Concentration (mg/mL) ^a^	PHS Concentration (mg/mL) ^a^
Alkaloids	4.32 ± 0.354	8.17 ± 1.13 *
Tannins	1.76 ± 0.287	3.54 ± 0.402 *
Flavonoids	6.11 ± 0.344	6.33 ± 0.392
β-Carotene	0.461 ± 0.075 *	0.194 ± 0.053
Lycopene	0.762 ± 0.138 *	0.136 ± 0.043

^a^ Values are means of triplicate determination (n = 3) ± standard deviations; * indicates significant difference (*p* < 0.05) between leaves and stem; PHL, *P. hydropiper* leaves; PHS, *P. hydropiper* stem.

**Table 2:** Antioxidant activity of *P*. *hydropiper* extracts in different solvents at different concentrations.

FRAP Activity	% Activity						
	Concentration (mg/mL) ^a^						
	0.46	0.94	1.88	3.75	7.50	15	IC_50_
PHL n-hexane	83.33 ± 7.2 **	79.28 ± 6.1 **	74.07 ± 3.5 **	63.86 ± 6.4 *	44.38 ± 11	32.87 ± 7.8 **	5.52
PHL acetone	74.72 ± 1.8 **	71.95 ± 4.1 **	51.53 ± 4.7 **	19.27 ± 3.1 **	8.43 ± 1.9	3.95 ± 0.8	2.29
PHL ethanol	68.37 ± 7.3 **	62.46 ± 3.2 **	56.68 ± 8.0 **	42.18 ± 4.3 **	29.35 ± 3.3	26.48 ± 4.8 *	2.99
PHL methanol	65.53 ± 3.2 **	59.78 ± 2.5 **	49.43 ± 3.8 **	25.97 ± 3.9 **	25.17 ± 3.3	12.87 ± 4.6	2.30
PHS n-hexane	72.15 ± 3.2 **	67.87 ± 5.3 **	45.94 ± 5.3 **	42.88 ± 1.6 *	31.45 ± 3.3	29.10 ± 6.1 *	1.50
PHS acetone	67.23 ± 12 **	52.70 ± 3.6 **	40.04 ± 6.5 **	16.03 ± 4.0 **	7.39 ± 1.4	3.57 ± 0.8 *	1.81
PHS ethanol	74.52 ± 3.32 **	69.66 ± 5.5 **	46.91 ± 5.1 **	43.67 ± 6.0 *	34.41 ± 4.8	28.66 ± 8.2	1.38
PHS methanol	69.31 ± 3.19 **	65.96 ± 5.3 **	44.33 ± 5.3 **	37.62 ± 4.0 **	27.61 ± 4.5	22.55 ± 6.0	1.73
**DPPH Activity**							
PHL acetone	72.91 ± 5.7	68.06 ± 3.6 **	65.16 ± 3.6 **	32.93 ± 6.0 *	22.03 ± 5.1 *	16.88 ± 2.2	2.94
PHL ethanol	82.68 ± 5.6	80.52 ± 4.4 **	72.02 ± 4.7 **	59.93 ± 5.6 **	42.19 ± 2.5	30.06 ± 1.3 *	5.14
PHS acetone	77.93 ± 3.2	71.95 ± 5.5 **	50 ± 5.1 **	46.21 ± 3.8 *	32.60 ± 3.3 *	27.57 ± 9.1	1.59
PHS ethanol	82.93 ± 9.4	78.13 ± 4.6 **	72.55 ± 5.8 **	64.78 ± 3.4 **	47.88 ± 0.2 *	36.72 ± 5.3 *	6.88

^a^ Values are means of triplicate determination (n = 3) ± standard deviations; *p* values less than 0.05 were considered to be statistically significant; * *p* < 0.05, ** *p* < 0.01; PHL, *P. hydropiper* leaves; PHS, *P. hydropiper* stem.

**Table 3:** Antimicrobial activity of different solvent extracts of *P. hydropiper* leaves and stem at different concentrations.

	Clear Zone of Inhibition (mm) of HP Extracts ^a^												Ant Ag (mm) ^a^	
Microorganisms	Leaf Concentration (mg/mL)						Stem Concentration (mg/mL)						DMSO	A
	0.46	0.94	1.88	3.75	7.50	15	0.46	0.94	1.88	3.75	7.50	15		
**Acetonic extract**														
*E. coli*	ND	ND	ND	ND	ND	ND	9	10	12	14	16	17	ND	20
*S. aureus*	ND	ND	ND	ND	ND	ND	9	11	13	15	17	18	ND	22
*K. pneumonia*	9	10	12	13	14	16	9	10	11	13	14	15	ND	17
*H. influenzae*	9	10	12	13	14	16	9	11	12	14	16	18	ND	20
*M. morganii*	10	12	13	14	15	17	10	12	14	15	17	19	ND	17
**Ethanolic extract**														
*E. coli*	9	10	11	13	14	15	9	11	12	13	14	16	ND	20
*S. aureus*	9	10	12	13	15	17	9	10	12	14	15	17	ND	22
*K. pneumonia*	7	8	9	13	14	15	9	10	12	13	14	16	ND	17
*H. influenzae*	7	11	12	14	15	17	10	11	12	13	15	17	ND	20
*M. morganii*	9	10	11	13	15	17	9	10	12	13	15	16	ND	17

^a^ Values are means of triplicate determination (n = 3); ND, not detected at this concentration; A, ampicillin (10 µg); DMSO, dimethyl sulfoxide (100%); Ant Ag, antimicrobial agent.
